# SHIFT-MAB: Fair and Mobility-Aware Handover Control for 6G Fully Decoupled RANs

**DOI:** 10.3390/s26082560

**Published:** 2026-04-21

**Authors:** Tian Gong, Chen Dai, Tongtong Yang

**Affiliations:** School of Computer Science, Nanjing University of Posts and Telecommunications, Nanjing 210023, China; b23041327@njupt.edu.cn (T.G.); 1225045434@njupt.edu.cn (T.Y.)

**Keywords:** fully decoupled radio access networks, multi-armed bandit, handover hysteresis, resource allocation, fairness, user mobility

## Abstract

Fully decoupled radio access networks (FD-RANs) achieve spectral efficiency and coverage flexibility for 6G via independent uplink (UL) and downlink (DL) base station operation, yet dynamic user mobility brings critical challenges to joint user association and resource allocation. Asymmetric interference and heterogeneous base station capacities cause persistent network unfairness, while uncoordinated mobility management triggers ping-pong handovers and heavy handover overheads. To resolve these intertwined problems, we propose a fully decoupled, mobility-resilient and fairness-guaranteed framework, which integrates short-term congestion pricing with the long-term Jain fairness index for equitable resource distribution and introduces a composite handover penalty with a strict physical hysteresis margin to block invalid handovers. We formulate the optimization problem as a novel Sliding-Window Hysteresis-Integrated Fairness Two-Layer Multi-Armed Bandit (SHIFT-MAB) model, embedding an exponentially weighted moving average (EWMA) sliding-window mechanism to track real-time channel fluctuations efficiently. Theoretical analysis confirms the model’s decoupling optimality, sublinear regret bound and fairness convergence. Extensive simulations show that SHIFT-MAB effectively suppresses invalid handovers, ensures high network fairness, optimizes system utility and achieves a superior handover–throughput trade-off.

## 1. Introduction

The evolution toward 6G wireless networks [1,2] promises unprecedented flexibility, ultra-low latency and massive connectivity [3,4]. To break through the performance bottlenecks of traditional coupled cellular architectures, the FD-RAN has emerged as a disruptive paradigm. By allowing UL and DL transmissions to associate with different base stations (BSs) independently, FD-RANs mitigate asymmetric UL/DL coverage disparities and significantly enhance the overall spectrum efficiency.

Despite such architectural merits, joint user association and resource allocation in FD-RANs face severe challenges under dynamic user mobility. Specifically, asymmetric interference and heterogeneous BS capacities inherently cause persistent network unfairness and severe congestion, as users greedily competing for optimal BSs can easily lead to unbalanced loads [5,6]. Furthermore, mobile users experience highly dynamic channel fading [7,8] and distance-dependent path loss, which frequently trigger handover (HO) events. While HOs are indispensable in sustaining link quality, uncoordinated mobility management often forces users to rapidly and redundantly switch back and forth between adjacent BSs. This phenomenon, widely known as the “ping-pong effect”, causes a prohibitive signaling overhead and disruptive service interruptions. Various intelligence-driven methods have been explored to alleviate these issues, including fuzzy logic-based vertical HO strategies [9] and machine learning-based HO failure prediction models [10].

Recently, DRL methods [11,12,13,14,15,16], such as Deep Q-Networks (DQNs) and Asynchronous Advantage Actor–Critic (A3C), have been widely adopted to solve HO and resource allocation problems [17,18,19,20]. For example, Cui et al. [18] successfully applied DRL to manage the massive combinatorial space of joint subchannel selection and power allocation. However, applying DRL directly to FD-RANs is highly problematic. First, the joint optimization of UL and DL associations inherently creates an exponentially large discrete action space, leading to the “curse of dimensionality”. Consequently, DRL agents require massive exploration episodes and struggle to converge in non-stationary environments caused by continuous user mobility. Second, traditional DRL architectures operate as “black boxes” that rely on soft penalties, making it difficult to strictly enforce physical constraints, such as HO hysteresis. This limitation often results in the aforementioned ping-pong HOs. On the other hand, conventional multi-armed bandit (MAB) algorithms fail to adapt to mobile environments because their infinite-memory mean estimation prevents them from tracking non-stationary channel dynamics [21,22].

To bridge these gaps and overcome the interconnected challenges of unfairness and redundant HOs, we propose a fully decoupled, mobility-resilient and fairness-guaranteed framework, namely Sliding-Window Hysteresis-Integrated Fairness Two-Layer MAB. By systematically integrating an explicit physical-layer hysteresis margin with a sliding-window bandit architecture, our proposed SHIFT-MAB physically blocks invalid HOs while maintaining high network fairness and achieving a superior HO–throughput trade-off.

The main contributions of this paper are summarized as follows:

- First, we formulate a joint UL/DL resource allocation problem in FD-RANs that rigorously captures dynamic mobility and asymmetric interference. Crucially, we introduce a composite handover cost model alongside a strict physical hysteresis margin to deterministically intercept redundant ping-pong HOs.
- To overcome the curse of dimensionality and the sluggish convergence of traditional DRL, we decouple the optimization into two parallel MABs for the UL and DL. Within this architecture, we embed a novel EWMA sliding-window mechanism, enabling decentralized agents to swiftly adapt to non-stationary channel fading.
- Furthermore, we design a fairness-aware utility function to implicitly coordinate decentralized agents without a prohibitive signaling overhead. This reward structure dynamically utilizes short-term congestion pricing to prevent base-station overloading while embedding Jain’s fairness index to guarantee long-term network equity.
- Finally, we theoretically prove the framework’s decoupling optimality and sublinear regret bound. Extensive simulations validate that SHIFT-MAB fundamentally resolves mobility bottlenecks, achieving a superior handover–throughput trade-off compared to state-of-the-art DRL baselines (e.g., D3QN and LSTM-A2C) across varied network densities and user velocities.

The remainder of this paper is organized as follows. Section 2 reviews the related work. Section 3 presents the system model, including mobility, channel and interference, handover and hysteresis and fairness metrics and utility design. Section 4 formulates the fairness-aware optimization problem. Section 5 develops the two-layer MAB formulation and the distributed mean index bandit algorithm and provides a theoretical analysis. Section 6 reports the simulation results and comparisons. Finally, we conclude this paper in Section 7.

## 2. Related Work

This section provides a summary of prior related work, focusing on the evolution of 6G architectures, resource allocation strategies and machine learning methods to ensure seamless mobility and fairness in highly dynamic wireless environments.

The transition toward 6G has driven a paradigm shift in radio access networks. Chen et al. [23] provided a comprehensive overview of the evolution of modern RANs, emphasizing the FD-RAN as a vital architecture to overcome the bottleneck of coupled UL and DL associations. In parallel, mobility management has been heavily explored to prevent session breakups and severe delays. Traditional mobility management frameworks, such as the distributed schemes proposed by Wagan and Jung [24] and the spectrum-aware protocols by Lee and Akyildiz [25], addressed core mobility issues but relied heavily on predefined heuristic rules. Similarly, Hoey et al. [26] explored mobility issues in low-power sensor networks, highlighting the significant energy and latency overheads caused by frequent handovers, while Goutam and Unnikrishnan [9] utilized fuzzy logic to optimize vertical handover decisions.

To address resource allocation in decoupled and dynamic environments, classical optimization techniques have been extensively explored. Qian et al. [27] proposed an elastic resource allocation framework for FD-RANs, utilizing matching algorithms and successive convex approximation (SCA) to dynamically share spectra and optimize power control. While these optimization-based protocols provide fine-grained control over resource assignments, they generally assume known and stationary system models with perfect or near-perfect channel state information (CSI). This limitation makes them computationally prohibitive and less suitable for the stochastic and time-varying channel conditions characteristic of high-mobility environments.

This limitation motivates the use of data-driven and model-free machine learning approaches. Early data-driven methods, such as the XGBoost model proposed by Manalastas et al. [10], demonstrated that supervised machine learning could effectively forecast inter-frequency handover failures. For continuous decision-making, DRL has proven to be a valuable tool. Zhao et al. [28] employed a D3QN to investigate joint user association and resource allocation in heterogeneous cellular networks, achieving excellent results in maximizing long-term utility. Similarly, Wang et al. [16] applied an A3C scheme to control handover processes, significantly lowering the handover rate while ensuring system throughput.

More recently, advanced learning-based methods have been adapted specifically to handle the scalability and non-stationarity of dynamic FD-RAN architectures. On the one hand, DRL approaches have demonstrated significant progress; for instance, Zhang et al. [29] presented a multi-agent deep reinforcement learning (MADRL) approach for inter-frequency multi-connectivity, while recent surveys and studies highlight the efficacy of modern MADRL variants [30] and federated multi-agent learning [31] in distributed sensing and spectrum management. Additionally, Chen et al. [32] proposed a stepwise RL approach to filter feasible actions in massive combinatorial spaces. However, when applied to mobility management, these DRL methods predominantly rely on reward shaping to handle constraints [16,28,29,32]. While effective for average performance, reward shaping does not guarantee strict physical constraints, as agents may still prefer immediate rewards over avoiding severe ping-pong handovers in edge cases. On the other hand, multi-armed bandit (MAB) algorithms provide a theoretically grounded alternative for non-stationary environments. Notably, Garivier and Moulines [33] established the foundations for sliding-window and discounted UCB, demonstrating their capacity to handle abrupt reward changes. Yet, while these modern bandit methods successfully address adaptability, they fundamentally focus on tracking reward estimations rather than enforcing structural network constraints.

Although the literature contains many improvements, there exists a significant research gap concerning the combination of decoupled association with explicit, hard hysteresis constraints to handle non-stationary mobility in FD-RAN systems. As shown in Table 1, while recent advanced learning approaches successfully improve adaptability, they often treat handover costs implicitly through soft penalty terms or lack the ability to adapt rapidly to the structural requirements of the network [16,27,28,29,32]. The purpose of this paper is to fill this gap by proposing SHIFT-MAB, a stochastic framework using an EWMA sliding-window mechanism combined with a strict physical hysteresis margin ($\delta_{\mathrm{hys}}$). Unlike standard sliding-window bandits [33] or DRL methods that rely on soft penalties, our approach explicitly integrates the non-stationary tracking ability of SW-MAB with a hard physical constraint to intercept ping-pong handovers, even under highly dynamic Gauss–Markov mobility conditions.

## 3. System Model

### 3.1. Network Model and Mobility Characterization

**Figure 1:**
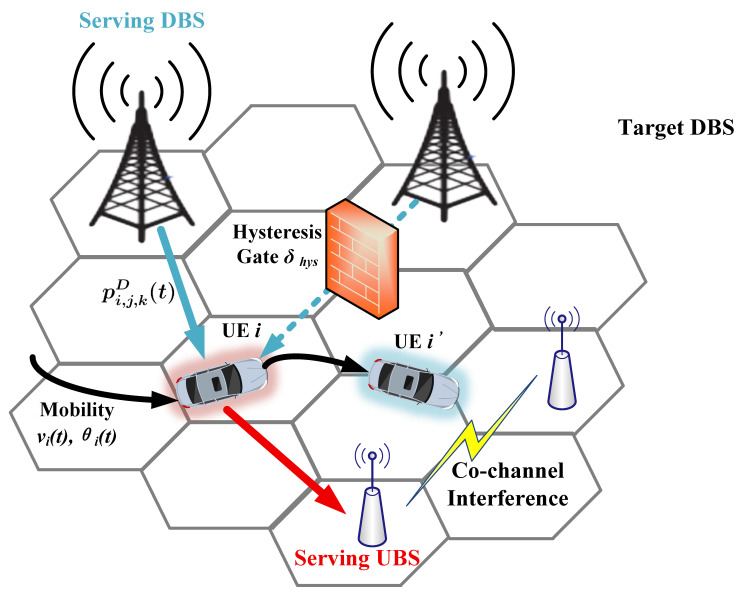
System model of the 6G FD-RAN scenario: mobile UE *i* with velocity $v_{i}\left(t\right)$ and direction $\theta_{i}\left(t\right)$, serving UL/DL base stations (UBS/DBS), target DBS for handover and the hysteresis gate $\delta_{\mathrm{hys}}$ that blocks ping-pong handovers.

We consider a 6G FD-RAN comprising *N* mobile user equipment (UE), $M^{U}$ uplink base stations (UBSs), $M^{D}$ downlink base stations (DBSs), $K^{U}$ orthogonal uplink channels and $K^{D}$ orthogonal downlink channels, where the superscript (·) denotes the transmission direction, with (·)=U for uplink and (·)=D for downlink. Let N={1,…,N} denote the UE set, $M^{U}=\left\{1,\ldots ,M^{U}\right\}$ the UBS set, $M^{D}=\left\{1,\ldots ,M^{D}\right\}$ the DBS set, $K^{U}=\left\{1,\ldots ,K^{U}\right\}$ the set of orthogonal uplink channels and $K^{D}=\left\{1,\ldots ,K^{D}\right\}$ the set of orthogonal downlink channels. Each BS *j* is deployed at a fixed 3D coordinate. $L_{j}={\left[x_{j},y_{j},z_{j}\right]}^{T}$, while each UE *i* has a time-varying position $P_{i}\left(t\right)={\left[x_{i}\left(t\right),y_{i}\left(t\right),z_{i}\left(t\right)\right]}^{T}$. The key notations used in this paper are summarized in Table 2 and the system model of the 6G fully-decoupled RAN is illustrated in Figure 1.

To capture realistic mobility patterns, we adopt the Gauss–Markov mobility model [34]. The velocity $v_{i}\left(t\right)$ and direction $\theta_{i}\left(t\right)$ of UE *i* evolve according to

(1) $$v_{i}\left(t\right)=\alpha v_{i}\left(t-1\right)+\left(1-\alpha \right)\bar{v}+\sqrt{1-\alpha^{2}}\;w_{v,t},$$

(2) $$\theta_{i}\left(t\right)=\alpha \theta_{i}\left(t-1\right)+\left(1-\alpha \right)\bar{\theta }+\sqrt{1-\alpha^{2}}\;w_{\theta ,t},$$

where α∈[0,1] is the memory parameter; $\bar{v}$ and $\bar{\theta }$ are the mean velocity and mean direction; and $w_{v,t}$, $w_{\theta ,t}$∼N(0,1) are independent Gaussian noise terms. The position is updated as

(3) $$\begin{array}{cc} x_{i}\left(t+1\right) & =x_{i}\left(t\right)+v_{i}\left(t\right)\cos\left(\theta_{i}\left(t\right)\right)\Delta t,\\ y_{i}\left(t+1\right) & =y_{i}\left(t\right)+v_{i}\left(t\right)\sin\left(\theta_{i}\left(t\right)\right)\Delta t,\\ z_{i}\left(t+1\right) & =z_{i}\left(t\right), \end{array}$$

where Δt is the discrete time step length, which corresponds to the slot duration. The vertical coordinate $z_{i}\left(t\right)$ remains constant, assuming ground-level or fixed-altitude mobility [35]. The total transmission rate of UE *i* in direction (·) at time *t* is obtained by aggregating over all associated BS–channel pairs:

(4) $$r_{i}^{\left(\cdot \right)}\left(t\right)=\sum_{j\in M^{\left(\cdot \right)}}\sum_{k\in K^{\left(\cdot \right)}}b_{i,j,k}^{\left(\cdot \right)}\left(t\right)\;r_{i,j,k}^{\left(\cdot \right)}\left(t\right),$$

where $b_{i,j,k}^{\left(\cdot \right)}\left(t\right)\in \left\{0,1\right\}$ is the binary association indicator, which equals 1 if UE *i* is associated with BS *j* on channel *k* in direction (·) at time *t* and 0 otherwise. The instantaneous Euclidean distance between UE *i* and BS *j* is

(5) $$d_{i,j}\left(t\right)={∥P_{i}\left(t\right)-L_{j}∥}_{2}.$$

### 3.2. Channel and Interference Model

The wireless channel between BS *j* and UE *i* on channel $k\in K^{\left(\cdot \right)}$ at time *t* is characterized by the instantaneous channel gain $h_{i,j,k}^{\left(\cdot \right)}\left(t\right)$, which incorporates both large-scale path loss and small-scale fading:

(6) $$h_{i,j,k}^{\left(\cdot \right)}\left(t\right)=\Omega \;d_{i,j}^{-\kappa }\left(t\right)\cdot {\left|\zeta_{i,j,k}\left(t\right)\right|}^{2},$$

where Ω is the path loss at a reference distance, κ is the path loss exponent and $|\zeta_{i,j,k}{\left(t\right)|}^{2}$ follows the Rayleigh fading distribution, reflecting multipath propagation.

In an FD-RAN, co-channel interference arises from simultaneous transmissions on the same frequency resource. For the uplink direction, the aggregate interference experienced by UE *i*’s signal at UBS *j* on channel *k* is generated by other UE transmitting on the same channel but associated with other BSs:

(7) $$I_{i,j,k}^{U}\left(t\right)=\sum_{j^{\prime }\in M^{U}\setminus \left\{j\right\}}\sum_{i^{\prime }\in N}b_{i^{\prime },j^{\prime },k}^{U}\left(t\right)\;p_{i^{\prime },j^{\prime },k}^{U}\left(t\right)\;h_{i^{\prime },j,k}^{U}\left(t\right),$$

where $b_{i^{\prime },j^{\prime },k}^{U}\left(t\right)\in \left\{0,1\right\}$ indicates whether UE $i^{\prime }$ is associated with UBS $j^{\prime }$ on channel *k*, and $p_{i^{\prime },j^{\prime },k}^{U}\left(t\right)$ is the transmit power of UE $i^{\prime }$ when associated with BS $j^{\prime }$ on channel *k*. Similarly, for the downlink direction, the interference at UE *i* on channel *k* when associated with BS *j* arises from neighboring BSs transmitting to their associated UE on the same channel:

(8) $$I_{i,j,k}^{D}\left(t\right)=\sum_{j^{\prime }\in M^{D}\setminus \left\{j\right\}}\sum_{i^{\prime }\in N}b_{i^{\prime },j^{\prime },k}^{D}\left(t\right)\;p_{i^{\prime },j^{\prime },k}^{D}\left(t\right)\;h_{i,j^{\prime },k}^{D}\left(t\right),$$

where $p_{i^{\prime },j^{\prime },k}^{D}\left(t\right)$ represents the transmit power of BS $j^{\prime }$ to UE $i^{\prime }$ on channel *k*. The signal-to-interference-plus-noise ratio (SINR) for UE *i* associated with BS *j* on channel *k* is

(9) $$\Gamma_{i,j,k}^{\left(\cdot \right)}\left(t\right)=\frac{p_{i,j,k}^{\left(\cdot \right)}\left(t\right)\;h_{i,j,k}^{\left(\cdot \right)}\left(t\right)}{I_{i,j,k}^{\left(\cdot \right)}\left(t\right)+W^{\left(\cdot \right)}N_{0}^{\left(\cdot \right)}},$$

where $W^{\left(\cdot \right)}$ is the channel bandwidth and $N_{0}^{\left(\cdot \right)}$ is the noise power spectral density. Note that the binary association indicator $b_{i,j,k}^{\left(\cdot \right)}\left(t\right)$ is omitted from the numerator of (9), as the power constraint in (23) strictly enforces $p_{i,j,k}^{\left(\cdot \right)}\left(t\right)=0$ whenever the association is inactive ($b_{i,j,k}^{\left(\cdot \right)}\left(t\right)=0$). The achievable transmission rate follows Shannon’s capacity formula:

(10) $$r_{i,j,k}^{\left(\cdot \right)}\left(t\right)=W^{\left(\cdot \right)}\log_{2}\;\left(1+\Gamma_{i,j,k}^{\left(\cdot \right)}\left(t\right)\right).$$

### 3.3. Handover Modeling and Cost Formulation

In an FD-RAN, HO events occur when UE switches its serving BS in either the UL or DL direction to maintain quality of service under dynamic channel conditions. Unlike conventional coupled systems, UL and DL handovers are triggered independently. The serving BS index for UE *i* at time *t* in direction (·) is determined from the association variables. If UE *i* is associated with BS *j* on channel *k* at time *t*, then $s_{i,t}^{\left(\cdot \right)}=j$, and the associated channel is $k_{i,t}^{\left(\cdot \right)}=k$. Formally, $s_{i,t}^{\left(\cdot \right)}=j$ and $k_{i,t}^{\left(\cdot \right)}=k$ if and only if $b_{i,j,k}^{\left(\cdot \right)}\left(t\right)=1$ for some $j\in M^{\left(\cdot \right)}$ and $k\in K^{\left(\cdot \right)}$. If no such association exists, we set $s_{i,t}^{\left(\cdot \right)}=0$ to indicate no active association. To prevent initial network access or reconnection from being incorrectly penalized as a handover event, the directional handover indicator is strictly defined as

(11) $$\chi_{i,t}^{\left(\cdot \right)}=\left\{\begin{array}{cc} 1, & \mathrm{if}\;s_{i,t}^{\left(\cdot \right)}\neq s_{i,t-1}^{\left(\cdot \right)}\;\mathrm{and}\;s_{i,t-1}^{\left(\cdot \right)}\neq 0,\\ 0, & \mathrm{otherwise}. \end{array}\right.$$

Each handover incurs both signaling overhead and service interruption. We model the composite handover cost for UE *i* in direction (·) at time *t* as

(12) $$E_{h,i}^{\left(\cdot \right)}\left(t\right)=E_{\mathrm{sig}}^{\left(\cdot \right)}+T_{\mathrm{ho}}^{\left(\cdot \right)}\cdot {\bar{r}}_{i,t-1}^{\left(\cdot \right)},$$

where $E_{\mathrm{sig}}^{\left(\cdot \right)}\geq 0$ is the energy consumed by RRC signaling messages exchanged during the handover preparation and execution phases in direction (·), and $T_{\mathrm{ho}}^{\left(\cdot \right)}\geq 0$ is the handover interruption time during which data transmission is suspended. The term ${\bar{r}}_{i,t-1}^{\left(\cdot \right)}$ denotes the EWMA of the user’s historical total rate defined in (4):

(13) $${\bar{r}}_{i,t}^{\left(\cdot \right)}=\beta_{r}\;{\bar{r}}_{i,t-1}^{\left(\cdot \right)}+\left(1-\beta_{r}\right)\;r_{i}^{\left(\cdot \right)}\left(t\right),$$

where $\beta_{r}\in \left[0,1\right]$ is the smoothing factor. The second term in (12) quantifies the opportunity cost of throughput loss during the interruption period, ensuring that frequent handovers are penalized proportionally to the sacrificed data rate.

To prevent the “ping-pong effect” caused by short-term channel fluctuations, we introduce a hysteresis margin $\delta_{\mathrm{hys}}>0$, which is a predefined positive constant measured in the same units as the transmission rate. A handover from serving BS $j_{\mathrm{old}}$ to target BS $j_{\mathrm{new}}$ is triggered only when the smoothed rate estimate for the target BS–channel combination exceeds that of the current serving BS by at least $\delta_{\mathrm{hys}}$. Specifically, let ${\bar{r}}_{i,j,k}^{\left(\cdot \right)}\left(t\right)$ denote the EWMA of the achievable rate $r_{i,j,k}^{\left(\cdot \right)}\left(t\right)$ when UE *i* is evaluated for BS *j* on channel *k*, updated as

(14) $${\bar{r}}_{i,j,k}^{\left(\cdot \right)}\left(t\right)=\beta_{r}\;{\bar{r}}_{i,j,k}^{\left(\cdot \right)}\left(t-1\right)+\left(1-\beta_{r}\right)\;r_{i,j,k}^{\left(\cdot \right)}\left(t\right).$$

In practical deployments, the UE continuously estimates the potential achievable rate $r_{i,j,k}^{\left(\cdot \right)}\left(t\right)$ of neighboring BSs via broadcast reference signals, even without maintaining an active data association. The handover trigger condition is

(15) $${\bar{r}}_{i,j_{\mathrm{new}},k}^{\left(\cdot \right)}\left(t\right)>{\bar{r}}_{i,j_{\mathrm{old}},k}^{\left(\cdot \right)}\left(t\right)+\delta_{\mathrm{hys}}.$$

This hysteresis mechanism ensures handover stability by requiring a sufficient quality improvement before switching.

### 3.4. Fairness Metrics and Utility Design

To ensure equitable resource distribution across all mobile users, we introduce Jain’s fairness index as a quantitative measure of long-term throughput equity [36]. At time *t*, the fairness index is defined as

(16) $$J\left(t\right)=\frac{{\left(\sum_{i=1}^{N}\left(r_{i}^{U}\left(t\right)+r_{i}^{D}\left(t\right)\right)\right)}^{2}}{N\sum_{i=1}^{N}{\left(r_{i}^{U}\left(t\right)+r_{i}^{D}\left(t\right)\right)}^{2}},$$

where $r_{i}^{U}\left(t\right)$ and $r_{i}^{D}\left(t\right)$ denote the UL and DL rates of UE *i* at time *t*. The index J(t)∈[1/N,1], where J(t)=1 indicates perfect fairness such that all users achieve equal rates, while J(t)→1/N reflects a severe disparity with one user monopolizing resources.

To mitigate short-term congestion, we introduce dynamic congestion prices for each BS *j* in direction (·). The price $\lambda_{j}^{\left(\cdot \right)}\left(t\right)$ reflects the resource scarcity and is updated via gradient ascent:

(17) $$\lambda_{j}^{\left(\cdot \right)}\left(t+1\right)=\max\left\{0,\;\lambda_{j}^{\left(\cdot \right)}\left(t\right)+\eta_{t}\left[\sum_{i=1}^{N}\sum_{k\in K^{\left(\cdot \right)}}b_{i,j,k}^{\left(\cdot \right)}\left(t\right)-C_{j}^{\left(\cdot \right)}\right]\right\},$$

where $\eta_{t}=\eta_{0}/\sqrt{t}$ is a diminishing step size, with $\eta_{0}>0$ being the initial step size, and $b_{i,j,k}^{\left(\cdot \right)}\left(t\right)\in \left\{0,1\right\}$ is the binary association indicator. Here, $C_{j}^{\left(\cdot \right)}\in Z_{+}$ serves as a soft capacity threshold for BS *j* in direction (·). Given the single-connectivity requirement in (21) and the orthogonal channel allocation in (22), BS *j* can physically serve at most $K^{\left(\cdot \right)}$ users simultaneously. Thus, setting $C_{j}^{\left(\cdot \right)}\leq K^{\left(\cdot \right)}$ establishes a load-balancing safety margin, triggering congestion prices before the physical channel resources are completely exhausted.

Integrating these components, we define the comprehensive utility function for UE *i* in direction (·) at time *t* as

(18) $$u_{i,t}^{\left(\cdot \right)}=r_{i}^{\left(\cdot \right)}\left(t\right)-\sum_{j\in M^{\left(\cdot \right)}}\sum_{k\in K^{\left(\cdot \right)}}b_{i,j,k}^{\left(\cdot \right)}\left(t\right)\;\lambda_{j}^{\left(\cdot \right)}\left(t\right)\;p_{i,j,k}^{\left(\cdot \right)}\left(t\right)+\omega \;J\left(t\right)-\beta_{\mathrm{ho}}\;E_{h,i}^{\left(\cdot \right)}\left(t\right)\;\chi_{i,t}^{\left(\cdot \right)},$$

where $r_{i}^{\left(\cdot \right)}\left(t\right)$ is the total transmission rate defined in (4), the second term penalizes resource usage at congested BSs weighted by the congestion price $\lambda_{j}^{\left(\cdot \right)}\left(t\right)$ and the transmit power $p_{i,j,k}^{\left(\cdot \right)}\left(t\right)$, the third term is the fairness reward with tunable weight ω>0 that scales the Jain’s index J(t), and the fourth term is the handover penalty [37] with tunable weight $\beta_{\mathrm{ho}}>0$ that scales the composite handover cost $E_{h}$ and indicator $\chi_{i,t}^{\left(\cdot \right)}$. This utility naturally balances individual performance, network-wide equity and mobility stability. Although the decision-making process is distributed across UE, the lightweight global scalars, namely the congestion prices $\lambda_{j}^{\left(\cdot \right)}\left(t\right)$ and the fairness index J(t), are periodically computed by the controller and broadcast to all UE via control channels. This mechanism enables implicit global coordination without incurring a prohibitive signaling overhead.

## 4. Problem Formulation

In 6G fully decoupled radio access networks, when performing resource allocation and mobility management for mobile users, it is essential to simultaneously consider both UL and DL transmissions while accounting for handover costs and fairness constraints. Given the independent operation of UL and DL base stations and the dynamic nature of wireless channels, employing a unified optimization framework that balances individual user performance, network-wide fairness and mobility stability becomes necessary for efficient resource utilization [38].

To this end, we formulate the fairness-aware joint UL-DL resource allocation problem as follows. At each time slot *t*, the aggregate system utility combines the utilities of all users across both transmission directions, weighted by factor γ∈[0,1], which reflects the relative importance of uplink versus downlink traffic:

(19) $$\Psi \left(t\right)=\sum_{i=1}^{N}\left(\gamma \;u_{i,t}^{U}+\left(1-\gamma \right)\;u_{i,t}^{D}\right),$$

where $u_{i,t}^{U}$ and $u_{i,t}^{D}$ denote the fairness-aware utilities defined in (18) for UE *i* in the uplink and downlink directions, respectively. The optimization objective is to maximize the expected cumulative system utility over an infinite time horizon:

(20) $$\max_{b_{i,j,k}^{\left(\cdot \right)},p_{i,j,k}^{\left(\cdot \right)}}\;E\left[\sum_{t=0}^{\infty }\rho^{t}\;\Psi \left(t\right)\right],$$

where ρ∈(0,1) is the temporal discount factor that reflects the network’s planning horizon and the degree to which future rewards are valued relative to immediate gains. The expectation is taken over the stochastic processes governing user mobility (5) and Rayleigh fading (6). Note that the congestion prices $\lambda_{j}^{\left(\cdot \right)}\left(t\right)$ are not optimization variables but rather auxiliary variables updated according to (17).

The decision variables in this formulation include the binary association indicator $b_{i,j,k}^{\left(\cdot \right)}\left(t\right)\in \left\{0,1\right\}$, which equals 1 if UE *i* is associated with BS *j* on channel *k* in direction (·) at time *t* and 0 otherwise. The transmit power level $p_{i,j,k}^{\left(\cdot \right)}\left(t\right)\in P^{\left(\cdot \right)}$ represents the power selected from the discrete power set $P^{\left(\cdot \right)}$ when UE *i* is associated with BS *j* on channel *k*. Additionally, $\lambda_{j}^{\left(\cdot \right)}\left(t\right)$ denotes the congestion price at BS *j* in direction (·) at time *t*, which evolves according to (17) and is not directly optimized but rather updated based on resource utilization.

Based on the above formulation, we can provide the following constraints that need to be satisfied:

(21) $$\sum_{j\in M^{\left(\cdot \right)}}\sum_{k\in K^{\left(\cdot \right)}}b_{i,j,k}^{\left(\cdot \right)}\left(t\right)\leq 1,\;\forall i\in N,\;\left(\cdot \right)\in \left\{U,D\right\},\;t,$$

(22) $$\sum_{i\in N}b_{i,j,k}^{\left(\cdot \right)}\left(t\right)\leq 1,\;\forall j\in M^{\left(\cdot \right)},\;k\in K^{\left(\cdot \right)},\;\left(\cdot \right)\in \left\{U,D\right\},\;t,$$

(23) $$0\leq p_{i,j,k}^{\left(\cdot \right)}\left(t\right)\leq P_{\max}^{\left(\cdot \right)}\;b_{i,j,k}^{\left(\cdot \right)}\left(t\right),\;\forall i\in N,\;j\in M^{\left(\cdot \right)},\;k\in K^{\left(\cdot \right)},\;\left(\cdot \right)\in \left\{U,D\right\},\;t,$$

(24) $$\chi_{i,t}^{\left(\cdot \right)}=1\Rightarrow {\bar{r}}_{i,s_{i,t}^{\left(\cdot \right)},k_{i,t}^{\left(\cdot \right)}}^{\left(\cdot \right)}\left(t\right)\geq {\bar{r}}_{i,s_{i,t-1}^{\left(\cdot \right)},k_{i,t-1}^{\left(\cdot \right)}}^{\left(\cdot \right)}\left(t\right)+\delta_{\mathrm{hys}},\;\forall i\in N,\;\left(\cdot \right)\in \left\{U,D\right\},\;t,$$

(25) $$\underset{T\to \infty }{lim inf}\frac{1}{T}\sum_{t=1}^{T}J\left(t\right)\geq J_{\min}.$$

Constraint (21) ensures that each user equipment is associated with at most one base station on a single channel per transmission direction at any time slot, reflecting the fundamental limitation of single-radio devices that cannot simultaneously maintain multiple active associations. Constraint (22) enforces channel orthogonality by stipulating that each frequency channel at a given base station serves at most one user equipment simultaneously, thereby avoiding intra-cell co-channel interference and maintaining the orthogonal multiple-access assumption underlying the SINR calculation in (9). The power budget constraint (23) bounds the transmit power level such that $p_{i,j,k}^{\left(\cdot \right)}\left(t\right)\leq P_{\max}^{\left(\cdot \right)}\;b_{i,j,k}^{\left(\cdot \right)}\left(t\right)$, ensuring that power is allocated only when an association is active, where $P_{\max}^{\left(\cdot \right)}$ represents either the maximum UE transmission power capability for uplink or the per-user power allocation limit at the base station for downlink, reflecting practical hardware limitations and regulatory spectral emission constraints. Constraint (24) introduces a hysteresis mechanism requiring that a handover event, which is indicated by $\chi_{i,t}^{\left(\cdot \right)}=1$, occurs only when the exponentially weighted moving average rate ${\bar{r}}_{i,s_{i,t}^{\left(\cdot \right)},k_{i,t}^{\left(\cdot \right)}}^{\left(\cdot \right)}\left(t\right)$ of the target base station–channel combination exceeds that of the current serving base station–channel combination ${\bar{r}}_{i,s_{i,t-1}^{\left(\cdot \right)},k_{i,t-1}^{\left(\cdot \right)}}^{\left(\cdot \right)}\left(t\right)$ by at least the hysteresis margin $\delta_{\mathrm{hys}}$, preventing ping-pong effects caused by short-term channel fluctuations. Finally, constraint (25) imposes a long-term fairness guarantee by requiring that the time-averaged Jain fairness index remains above a minimum threshold $J_{\min}\in \left[1/N,1\right]$, which is a predefined parameter chosen by the network operator to ensure that no user experiences persistent performance degradation.

However, the resulting optimization problem is intrinsically difficult to solve due to several factors. First, the binary association and channel allocation variables lead to a massive combinatorial action space of size $O\left({\left(M^{U}M^{D}K^{U}K^{D}L^{2}\right)}^{N}\right)$ for joint UL and DL decisions. Such an exponentially large discrete-continuous hybrid space is notoriously difficult to optimize, as similarly observed in large-scale multi-user resource scheduling [14,17]. Moreover, the fairness-aware utility function (18) exhibits severe non-convexity due to the logarithmic rate function (10) coupled with the SINR formula (9), the rational Jain fairness index (16) and the dynamic congestion prices (17). Furthermore, the handover penalty indicator $\chi_{i,t}^{\left(\cdot \right)}$ and the hysteresis constraint (24) introduce strict temporal coupling across consecutive decision epochs. This temporal dependency, combined with the non-stationary channel fading and the curse of dimensionality, renders traditional static convex optimization methods computationally intractable. Therefore, it is imperative to reformulate the problem into a sequential and decentralized learning framework, naturally motivating the proposed SHIFT-MAB architecture detailed in the following section.

## 5. The Proposed SHIFT-MAB Framework

To address the computational challenges inherent in the fairness-aware optimization problem formulated in Section 4, we develop a distributed learning framework [39,40] that reformulates the joint UL-DL association and resource allocation task as an MAB problem. This reformulation enables each UE to independently learn optimal strategies through local observations while maintaining coordination through shared pricing and fairness signals.

### 5.1. Decoupled Two-Layer Bandit Architecture

To enable scalable and distributed decision-making, the system-level optimization problem in (20) is decomposed into two directionally separated subproblems [41], corresponding to UL and DL, respectively:

(26) $$\max_{b^{\left(U\right)},\;p^{\left(U\right)}}\;E\left[\sum_{t=0}^{\infty }\rho^{t}\sum_{i=1}^{N}\gamma \;u_{i,t}^{U}\right],$$

(27) $$\max_{b^{\left(D\right)},\;p^{\left(D\right)}}\;E\left[\sum_{t=0}^{\infty }\rho^{t}\sum_{i=1}^{N}\left(1-\gamma \right)\;u_{i,t}^{D}\right],$$

where $u_{i,t}^{U}$ and $u_{i,t}^{D}$ denote the UL and DL utilities of UE *i* at time *t* as defined in (18), respectively. This decomposition is consistent with the FD-RAN architecture, in which UL and DL operate on orthogonal spectra with different BS sets, and the interference patterns are directionally separable [7,18].

The decoupling strategy reduces the computational complexity from $O(N^{2}|A{|}^{2})$ to $O(N(|A^{U}\left|+\right|A^{D}\left|)\right)$, where $\left|A\right|=M^{U}\times M^{D}\times K^{U}\times K^{D}\times L^{2}$ represents the joint action space size. Under the structural independence of UL and DL base stations in the FD-RAN, this decomposition preserves optimality, as interference patterns and pricing mechanisms are separable and the coupling is maintained through shared prices $\lambda_{j}^{\left(\cdot \right)}\left(t\right)$ and the global fairness index J(t).

To operationalize this decomposition, we present Algorithm 1, which formalizes how the joint UL-DL problem is separated into independent subproblems while preserving coupling through shared pricing and fairness signals.
**Algorithm 1** FD-RAN UL-DL Decoupling 1:**Input:** Network topology; UE set N; BS sets $M^{U}$, $M^{D}$; channel sets $K^{U}$, $K^{D}$; 2:**Output:** Decoupled action spaces $A_{i}^{U}$, $A_{i}^{D}$; independent reward functions $R_{i}^{U}$, $R_{i}^{D}$; 3:**Step 1: Network Decomposition**; 4:**for all** UE i∈N **do** 5: Extract UL action space: $A_{i}^{U}=\left\{\left(j,k,p\right):j\in M^{U},k\in K^{U},p\in P^{U}\right\}$; 6: Extract DL action space: $A_{i}^{D}=\left\{\left(j,k,p\right):j\in M^{D},k\in K^{D},p\in P^{D}\right\}$; 7:**end for** 8:**Step 2: Independent Interference Modeling**; 9:Calculate UL interference: $I_{i,j,k}^{U}\left(t\right)=\sum_{j^{\prime }\neq j}\sum_{i^{\prime }}b_{i^{\prime },j^{\prime },k}^{U}\left(t\right)p_{i^{\prime },j^{\prime },k}^{U}\left(t\right)h_{i^{\prime },j,k}^{U}\left(t\right)$ (cf. (7));10:Calculate DL interference: $I_{i,j,k}^{D}\left(t\right)=\sum_{j^{\prime }\neq j}\sum_{i^{\prime }}b_{i^{\prime },j^{\prime },k}^{D}\left(t\right)p_{i^{\prime },j^{\prime },k}^{D}\left(t\right)h_{i,j^{\prime },k}^{D}\left(t\right)$ (cf. (8));11:**Step 3: Decoupled Reward Computation**;12:Compute UL reward: $R_{i}^{U}\left(t;a\right)=r_{i}^{U}\left(t\right)-\lambda_{j}^{U}\left(t\right)p_{i,j,k}^{U}+\omega J\left(t\right)-\beta_{\mathrm{ho}}E_{h,i}^{U}\left(t\right)\chi_{i,t}^{U}$;13:Compute DL reward: $R_{i}^{D}\left(t;a\right)=r_{i}^{D}\left(t\right)-\lambda_{j}^{D}\left(t\right)p_{i,j,k}^{D}+\omega J\left(t\right)-\beta_{\mathrm{ho}}E_{h,i}^{D}\left(t\right)\chi_{i,t}^{D}$;14:**Return**${\left\{A_{i}^{U},A_{i}^{D},R_{i}^{U},R_{i}^{D}\right\}}_{i\in N}$;

### 5.2. Multi-Agent Bandit Formulation

Leveraging the decomposition in (26) and (27), the resource allocation task is modeled as a multi-agent multi-armed bandit (MAMAB) framework, where each user acts as an autonomous learning agent that adaptively selects its UL and DL association strategies based on local observations, and the dynamic price signals $\lambda_{j}^{\left(\cdot \right)}\left(t\right)$ provide lightweight coordination to align individual decisions with global fairness objectives.

For each transmission direction (·)∈{U,D}, the action space of UE *i* is defined as a tuple consisting of the BS association, channel selection and power level:

(28) $$A_{i}^{\left(\cdot \right)}=M^{\left(\cdot \right)}\times K^{\left(\cdot \right)}\times P^{\left(\cdot \right)},$$

where $M^{\left(\cdot \right)}$ represents the candidate BS set, $K^{\left(\cdot \right)}$ the available channels and $P^{\left(\cdot \right)}$ the discrete power level set. At each decision epoch *t*, UE *i* chooses one action,

(29) $$a_{i,t}^{\left(\cdot \right)}=\left(j^{\left(\cdot \right)},k^{\left(\cdot \right)},p^{\left(\cdot \right)}\right)\in A_{i}^{\left(\cdot \right)},$$

which corresponds to the BS association $j^{\left(\cdot \right)}$, channel selection $k^{\left(\cdot \right)}$ and power level $p^{\left(\cdot \right)}$ in the given direction. Given an action $a_{i,t}^{\left(\cdot \right)}$, the instantaneous reward is defined as the fairness-aware utility:

(30) $$R_{i}^{\left(\cdot \right)}\left(t;a_{i,t}^{\left(\cdot \right)}\right)=u_{i,t}^{\left(\cdot \right)},$$

where $u_{i,t}^{\left(\cdot \right)}$ integrates throughput $r_{i}^{\left(\cdot \right)}\left(t\right)$, congestion penalties weighted by $\lambda_{j}^{\left(\cdot \right)}\left(t\right)$, fairness reward ωJ(t) and handover penalty $\beta_{\mathrm{ho}}\;E_{h,i}^{\left(\cdot \right)}\left(t\right)\;\chi_{i,t}^{\left(\cdot \right)}$ as defined in (18). Thus, the reward simultaneously reflects local efficiency and system-level fairness. To maintain consistency between UL and DL decisions, both directional rewards are regulated through shared fairness signals:

(31) $$R_{i}^{\left(\cdot \right)}\left(t\right)=f^{\left(\cdot \right)}\;\left(a_{i,t}^{\left(\cdot \right)},\lambda^{\left(\cdot \right)}\left(t\right),J\left(t\right),E_{h,i}^{\left(\cdot \right)}\left(t\right)\right),$$

where $f^{\left(\cdot \right)}\left(\cdot \right)$ is the direction-specific fairness-aware reward function incorporating congestion prices, global fairness index and handover costs. The shared pricing and fairness terms implicitly coordinate users’ independent decisions, guiding them toward overall network efficiency and balanced performance.

### 5.3. SHIFT-MAB Algorithm and Theoretical Guarantees

**Figure 2:**
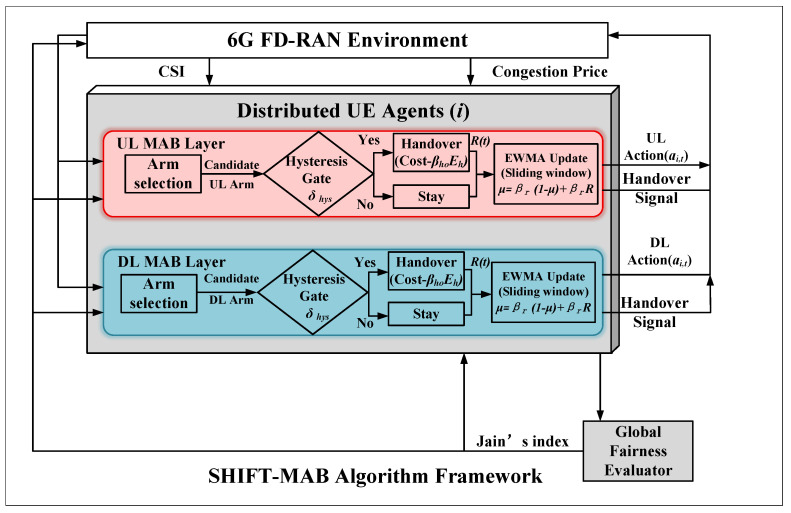
SHIFT-MAB algorithm framework: distributed UE agents with UL and DL MAB layers, hysteresis gate $\delta_{\mathrm{hys}}$ in arm selection, EWMA sliding-window update and global fairness evaluator (Jain’s index). Inputs: CSI and congestion price λ. Outputs: actions $a_{i,t}$ and handover signals $\chi_{i,t}$.

Building upon the decoupled action spaces from Algorithm 1, we propose the SHIFT-MAB framework, detailed in Algorithm 2. Unlike conventional bandits that rely on infinite-memory averaging, SHIFT-MAB utilizes an EWMA to create a soft sliding window, enabling the agent to swiftly adapt to non-stationary channel fading induced by Gauss–Markov mobility. Furthermore, a physical hysteresis margin is strictly integrated into the arm selection policy to intercept suboptimal ping-pong handovers deterministically. Figure 2 illustrates the overall SHIFT-MAB architecture.
**Algorithm 2** SHIFT-MAB: Sliding-Window Hysteresis-Integrated Fairness Two-Layer MAB**Input:** 
Decoupled spaces $A_{i}^{U},A_{i}^{D}$ from Algorithm 1; Exploration budget *P*; EWMA factor $\beta_{r}$ (as in (13)); Hysteresis margin $\delta_{\mathrm{hys}}$; Fairness weight ω; Penalty weight $\beta_{\mathrm{ho}}$.
**Output:** 
Optimal associated arms $a_{i,t}^{U*},a_{i,t}^{D*}$ and updated system utilities.
 1:**Initialization:** $\forall a\in A_{i}^{\left(\cdot \right)}$, set play count $o_{i}^{\left(\cdot \right)}\left(a\right)\leftarrow 0$, mean estimate ${\hat{\mu }}_{i}^{\left(\cdot \right)}\left(a\right)\leftarrow 0$. Initialize congestion prices $\lambda_{j}^{\left(\cdot \right)}\left(1\right)\leftarrow 0$, historical rates ${\bar{r}}_{i,j,k}^{\left(\cdot \right)}\left(0\right)\leftarrow 0$. 2:**for** time slot t=1,2,…,T **do** 3:     Update UE positions $P_{i}\left(t\right)$ via Gauss–Markov mobility (1)–(3); refresh channel gains $h_{i,j,k}^{\left(\cdot \right)}\left(t\right)$. 4:     **for** each direction (·)∈{U,D} and each UE i∈N **in parallel do** 5:          **if** $\exists a\in A_{i}^{\left(\cdot \right)}$ such that $o_{i}^{\left(\cdot \right)}\left(a\right)<P$ **then** 6:              **[Exploration]** Select underexplored arm *a*. 7:          **else** 8:              **[Exploitation and Hysteresis Integration]** 9:              Find candidate arm: $a_{\mathrm{cand}}\leftarrow \arg\max_{a}{\hat{\mu }}_{i}^{\left(\cdot \right)}\left(a\right)$.10:             Let $a_{\mathrm{curr}}$ be the arm associated at t−1.11:             **if** ${\bar{r}}_{i}^{\left(\cdot \right)}\left(a_{\mathrm{cand}}\right)>{\bar{r}}_{i}^{\left(\cdot \right)}\left(a_{\mathrm{curr}}\right)+\delta_{\mathrm{hys}}$ **then**12:                 $a_{i,t}^{(\cdot )*}\leftarrow a_{\mathrm{cand}}$13:             **else**14:                 $a_{i,t}^{(\cdot )*}\leftarrow a_{\mathrm{curr}}$15:             **end if**16:             Play selected arm $a_{i,t}^{(\cdot )*}$ and observe reward $R_{i}^{\left(\cdot \right)}\left(t\right)$ from (30).17:          **end if**18:          **[Sliding-Window Update]**19:          $o_{i}^{\left(\cdot \right)}\left(a_{i,t}^{(\cdot )*}\right)\leftarrow o_{i}^{\left(\cdot \right)}\left(a_{i,t}^{(\cdot )*}\right)+1$20:          ${\hat{\mu }}_{i}^{\left(\cdot \right)}\left(a_{i,t}^{(\cdot )*}\right)\leftarrow \left(1-\beta_{r}\right){\hat{\mu }}_{i}^{\left(\cdot \right)}\left(a_{i,t}^{(\cdot )*}\right)+\beta_{r}R_{i}^{\left(\cdot \right)}\left(t\right)$21:     **end for**22:     Calculate actual system utility by deducting handover penalty $\beta_{\mathrm{ho}}E_{h,i}^{\left(\cdot \right)}\left(t\right)\chi_{i,t}^{\left(\cdot \right)}$; update $\lambda_{j}^{\left(\cdot \right)}\left(t+1\right)$ via (17) and J(t) via (16).23:**end for**


#### 5.3.1. SHIFT-MAB Algorithm Design

At each time step, UE *i* maintains, for each arm $a\in A_{i}^{\left(\cdot \right)}$, the cumulative reward $s_{i}^{\left(\cdot \right)}\left(a\right)$ and play count $o_{i}^{\left(\cdot \right)}\left(a\right)$. The mean reward estimate is

(32) $${\hat{\mu }}_{i}^{\left(\cdot \right)}\left(a\right)=\frac{s_{i}^{\left(\cdot \right)}\left(a\right)}{o_{i}^{\left(\cdot \right)}\left(a\right)},$$

where $s_{i}^{\left(\cdot \right)}\left(a\right)$ is the sum of rewards observed when arm *a* was played and $o_{i}^{\left(\cdot \right)}\left(a\right)$ is the number of times that *a* was played. Each arm is sampled *P* times during exploration; once $o_{i}^{\left(\cdot \right)}\left(a\right)\geq P$ for all arms, the algorithm selects the arm with the highest mean index, i.e., $a_{i,t}^{(\cdot )*}=\arg\max_{a\in A_{i}^{\left(\cdot \right)}}{\hat{\mu }}_{i}^{\left(\cdot \right)}\left(a\right)$. It is worth noting that, during this initial pure exploration phase, the algorithm temporarily bypasses the hysteresis margin $\delta_{\mathrm{hys}}$ to ensure sufficient sampling of unknown channels. Since the exploration budget *P* is extremely small, the overhead induced by these brief forced handovers is negligible and perfectly traded for long-term stability. In Algorithm 2, the estimate ${\hat{\mu }}_{i}^{\left(\cdot \right)}\left(a\right)$ is updated via an EWMA for non-stationary adaptation; the theoretical bounds below are stated for the sample-mean statistic (32), which coincides with the EWMA in the limit $\beta_{r}\to 1$ and for which concentration holds. The true expected reward of arm *a* is $\mu_{i}^{\left(\cdot \right)}\left(a\right)=E\left[R_{i}^{\left(\cdot \right)}\left(t;a\right)\right]$. The deviation between the sample mean ${\hat{\mu }}_{i}^{\left(\cdot \right)}\left(a\right)$ and $\mu_{i}^{\left(\cdot \right)}\left(a\right)$ satisfies

(33) $$|{\hat{\mu }}_{i}^{\left(\cdot \right)}\left(a\right)-\mu_{i}^{\left(\cdot \right)}\left(a\right)|\leq \sqrt{\frac{2\log T}{o_{i}^{\left(\cdot \right)}\left(a\right)}}+\frac{1}{\sqrt{o_{i}^{\left(\cdot \right)}\left(a\right)}},$$

with probability at least $1-T^{-2}$, under bounded rewards.

#### 5.3.2. Decoupling Optimality

The congestion price $\lambda_{j}^{\left(\cdot \right)}\left(t\right)$ is updated via (17) with $\eta_{t}=\eta_{0}/\sqrt{t}$, and the global fairness index J(t) is given by (16). The handover cost $E_{h,i}^{\left(\cdot \right)}\left(t\right)$ and indicator $\chi_{i,t}^{\left(\cdot \right)}$ follow (12) and (11), so that the utility (18) penalizes congestion and handovers while rewarding fairness. The algorithm has complexity $O(N\cdot |A_{i}^{\left(\cdot \right)}|\cdot P)$ per time step, where

(34) $$|A_{i}^{\left(\cdot \right)}|=M^{\left(\cdot \right)}\times K^{\left(\cdot \right)}\times L,$$

and is scalable to large FD-RAN deployments.

The decomposition in Algorithm 1 independently optimizes UL and DL resource selection. Under the structural independence of FD-RANs, where UL and DL operate on orthogonal spectra and are served by different BS sets, the action space is reduced from $|A_{i}^{U}\left|\;\times \;\right|A_{i}^{D}\;|\;$ to $|A_{i}^{U}\left|\;+\;\right|A_{i}^{D}|$ while preserving optimality.

**Theorem** **1** (Decoupling Optimality Preservation). *Under the structural independence of the FD-RAN, where UL and DL use orthogonal frequency bands, for any time t and UE i, the optimal joint action $\left(a_{i,t}^{U*},a_{i,t}^{D*}\right)$ satisfies*

(35) $$\arg\max_{\left(a^{U},a^{D}\right)\in A_{i}^{U}\times A_{i}^{D}}E\left[R_{i}\left(a^{U},a^{D},t\right)\right]=\left(\arg\max_{a^{U}\in A_{i}^{U}}E\left[R_{i}^{U}\left(t;a^{U}\right)\right],\;\arg\max_{a^{D}\in A_{i}^{D}}E\left[R_{i}^{D}\left(t;a^{D}\right)\right]\right),$$

*where the joint reward is*

(36) $$R_{i}\left(a^{U},a^{D},t\right)=\gamma \;R_{i}^{U}\left(t;a^{U}\right)+\left(1-\gamma \right)\;R_{i}^{D}\left(t;a^{D}\right).$$

**Proof.**  From (30) and (18), the joint reward at time *t* is $R_{i}\left(a^{U},a^{D},t\right)=\gamma \;u_{i,t}^{U}\left(a^{U}\right)+\left(1-\gamma \right)\;u_{i,t}^{D}\left(a^{D}\right)$. UL and DL use orthogonal bands and separate BS sets, so interference and pricing are directionally separable. At time *t*, J(t) is the same for all action pairs $\left(a^{U},a^{D}\right)$ of UE *i*. Hence,

$$\arg\max_{\left(a^{U},a^{D}\right)}[\gamma R_{i}^{U}\left(t;a^{U}\right)+\left(1-\gamma \right)R_{i}^{D}\left(t;a^{D}\right)]=\left(\arg\max_{a^{U}}R_{i}^{U}\left(t;a^{U}\right),\;\arg\max_{a^{D}}R_{i}^{D}\left(t;a^{D}\right)\right),$$

and Algorithm 1 preserves optimality by optimizing each direction independently.  □

#### 5.3.3. Regret and Mean Index Convergence

We next characterize the learning performance of the proposed mean index policy from a regret perspective. For each UE *i* and direction (·)∈{U,D}, the expected reward of arm $a\in A_{i}^{\left(\cdot \right)}$ and the optimal value are defined as

(37) $$\begin{array}{cc} \mu_{i}^{\left(\cdot \right)}\left(a\right) & =E\left[R_{i}^{\left(\cdot \right)}\left(t;a\right)\right],\\ \mu_{i}^{(\cdot )*} & =\max_{a\in A_{i}^{\left(\cdot \right)}}\mu_{i}^{\left(\cdot \right)}\left(a\right). \end{array}$$

The cumulative regret over horizon *T* is defined as

(38) $$\rho_{i}^{\left(\cdot \right)}\left(T\right)=T\mu_{i}^{(\cdot )*}-\sum_{t=1}^{T}E\left[R_{i}^{\left(\cdot \right)}\left(t;a_{i,t}^{\left(\cdot \right)}\right)\right],$$

which measures the performance loss of the learning policy relative to always playing the best arm.

The mean index policy in Algorithm 2 operates in two phases. In the *pure exploration* phase, each arm is sampled at most *P* times to obtain an initial estimate (32). In the subsequent *exploitation* phase, the UE selects, at each slot, the arm with the largest empirical mean:

(39) $$a_{i,t}^{(\cdot )*}=\arg\max_{a\in A_{i}^{\left(\cdot \right)}}{\hat{\mu }}_{i}^{\left(\cdot \right)}\left(a\right).$$

The concentration bound in (33) implies that, as the play count $o_{i}^{\left(\cdot \right)}\left(a\right)$ grows, the deviation between ${\hat{\mu }}_{i}^{\left(\cdot \right)}\left(a\right)$ and $\mu_{i}^{\left(\cdot \right)}\left(a\right)$ shrinks at a rate $O\left(\sqrt{\log T/o_{i}^{\left(\cdot \right)}\left(a\right)}\right)$ with a high probability, so suboptimal arms are pulled only a logarithmic number of times in expectation.

**Proposition** **1** (Regret Bound for Mean Index Policy). *For each UE i and direction (·), let an optimal arm and the reward gaps be defined as*

(40) $$\begin{array}{cc} a^{*}= & \arg\max_{a\in A_{i}^{\left(\cdot \right)}}\mu_{i}^{\left(\cdot \right)}\left(a\right),\\ \Delta_{a}^{\left(\cdot \right)} & =\mu_{i}^{(\cdot )*}-\mu_{i}^{\left(\cdot \right)}\left(a\right),\\ & \Delta_{\max}^{\left(\cdot \right)}=\max_{a\neq a^{*}}\Delta_{a}^{\left(\cdot \right)}. \end{array}$$

*Under the mean index policy with exploration budget P and sample-mean estimates (32), the expected cumulative regret satisfies*

(41) $$E\left[\rho_{i}^{\left(\cdot \right)}\left(T\right)\right]\;\leq \;\left|A_{i}^{\left(\cdot \right)}\right|\cdot P\cdot \Delta_{\max}^{\left(\cdot \right)}\;+\;C_{1}\;\sum_{a\neq a^{*}}\frac{\log T}{\Delta_{a}^{\left(\cdot \right)}},$$

*for some constant $C_{1}>0$ independent of T (depending on the reward scale). Consequently, $E\left[\rho_{i}^{\left(\cdot \right)}\left(T\right)\right]=O(|A_{i}^{\left(\cdot \right)}\left|\log T\right)$ is sublinear in T, and the mean index policy is asymptotically optimal in the sense that*

(42) $$\lim_{T\to \infty }\frac{E[\rho_{i}^{\left(\cdot \right)}\left(T\right)]}{T}=0.$$

**Proof.**  During pure exploration, each arm is played at most *P* times, so the corresponding regret is at most $|A_{i}^{\left(\cdot \right)}|\cdot P\cdot \Delta_{\max}^{\left(\cdot \right)}$. In the exploitation phase, the policy chooses $a_{i,t}^{(\cdot )*}=\arg\max_{a}{\hat{\mu }}_{i}^{\left(\cdot \right)}\left(a\right)$. By applying Hoeffding’s inequality for bounded rewards, we can explicitly define a confidence parameter δ∈(0,1) to bound the estimation error. For an arm *a* pulled $o_{i}^{\left(\cdot \right)}\left(a\right)$ times, the concentration inequality is given by

(43) $$P(|\hat{\mu }-\mu \left|\geq \epsilon \right)\leq 2\exp\left(-2o_{i}^{\left(\cdot \right)}\left(a\right)\epsilon^{2}\right).$$

By setting the error margin as $\epsilon =\sqrt{\frac{1}{2o_{i}^{\left(\cdot \right)}\left(a\right)}\ln\left(\frac{2}{\delta }\right)}$, this guarantees that $|\hat{\mu }-\mu |<\epsilon$ holds with a high probability of at least 1−δ. In our framework, by linking the confidence bound to the time horizon as $\delta =O(T^{-2})$, the event of falsely preferring a suboptimal arm *a* requires the estimation error to exceed half of the reward gap, which decays exponentially as $O(\exp\left(-2\;\Delta_{a}^{(\cdot )2}o_{i}^{\left(\cdot \right)}\left(a\right)\right))$. Therefore, to strictly bound this error probability to $O(T^{-2})$, the required number of pulls for the suboptimal arm must reach exactly $o_{i}^{\left(\cdot \right)}\left(a\right)=O\left(\log T/\Delta_{a}^{(\cdot )2}\right)$. Hence, the expected number of pulls of arm *a* in the exploitation phase is bounded by $O(\log T/\Delta_{a}^{(\cdot )2})$, contributing at most $O\left(\Delta_{a}^{\left(\cdot \right)}\cdot \log T/\Delta_{a}^{(\cdot )2}\right)=O\left(\log T/\Delta_{a}^{\left(\cdot \right)}\right)$ to the regret. Summing over $a\neq a^{*}$ yields the second term in (41).  □

Aggregating over all UEs and both transmission directions, the network-wide cumulative regret satisfies

(44) $$\sum_{i=1}^{N}\left(\rho_{i}^{U}\left(T\right)+\rho_{i}^{D}\left(T\right)\right)=O\left(N\;|A_{i}^{\left(\cdot \right)}|\log T\right),$$

implying that the proposed SHIFT-MAB framework achieves sublinear system-level regret while preserving the UL/DL decoupling structure.

#### 5.3.4. Fairness Convergence

Under the price-based mechanism (17) and the fairness reward ωJ(t) in (18), the time-averaged Jain index is driven toward a high-fairness region.

**Remark** **1** (Fairness Convergence Mechanism). *Assume that the congestion price $\lambda_{j}^{\left(\cdot \right)}\left(t\right)$ is updated via (17) with a diminishing step size $\eta_{t}=\eta_{0}/\sqrt{t}$, and the utility incorporates the fairness term ωJ(t) with ω>0. Under standard stochastic approximation and ergodicity conditions on the reward process, the time-averaged Jain index is expected to satisfy*

(45) $$\underset{T\to \infty }{lim inf}\frac{1}{T}\sum_{t=1}^{T}J\left(t\right)\geq J_{\min},$$

*where $J_{\min}\in \left[1/N,1\right]$ is the minimum required fairness threshold defined in (25).*

*Intuitively, the dynamic congestion pricing penalizes overloaded BSs and encourages users to choose underutilized resources. Meanwhile, the explicit reward term ωJ(t) directly incentivizes balanced data rates across the network. Because the mean index policy continuously drives arm selection toward higher-reward actions, this coupled reward structure empirically pushes the network’s empirical rate distribution toward more balanced allocation over time, enabling the system to fulfill the long-term fairness constraint (45) without requiring a prohibitive signaling overhead.*

## 6. Simulation Results and Analysis

### 6.1. Experimental Setup

Simulations are implemented using Python 3.13 and conducted in an FD-RAN environment. The coverage area is 100m×100m (horizontal), with BS and UE heights set to 25 m and 1.5 m; user mobility follows the Gauss–Markov model with a time step of Δt=1s and a memory factor of 0.7. Unless otherwise stated, the default scenario consists of N=5 UE, $M^{U}=M^{D}=3$ uplink and downlink base stations, $K^{U}=K^{D}=3$ orthogonal channels per direction and L=5 discrete power levels ranging from 0.5W to 2.5W per level. The total number of iterations is set to T=4000. The path loss exponent is 2, the noise power spectral density is set to −174dBm/Hz, and the bandwidth is 20MHz per link. The proposed fairness-aware bandit uses a mean index exploration strategy with an adaptive sampling interval *P*, neighbor-based candidate sampling and a comprehensive reward function that embeds Jain’s fairness index alongside a handover penalty term characterized by $\beta_{\mathrm{ho}}$. The congestion price is updated with an initial step $\eta_{0}=10$ and is capped at $\lambda_{\max}=5$. The fairness weight in the reward is set to ω=300. The key system and algorithm parameters are summarized in Table 3.

### 6.2. Convergence and Scalability Analysis

**Figure 3:**
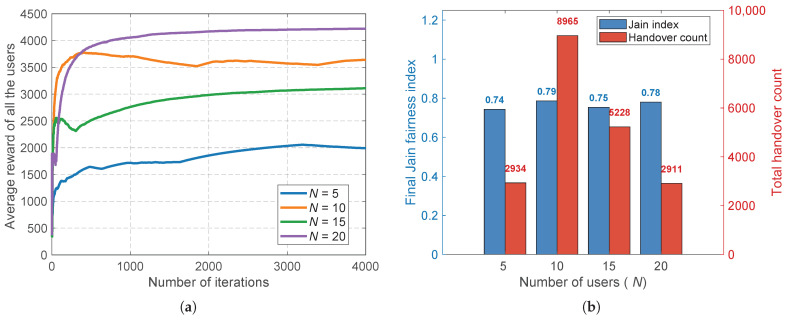
Scalability evaluation with respect to the number of users *N*. (**a**) Convergence of the average reward for all users, demonstrating robust learning efficiency across varying densities. (**b**) The resilience of the final Jain’s fairness index and the controlled growth of the total handover count as the network scales.

To evaluate the learning efficiency and structural scalability of the proposed SHIFT-MAB framework, we first investigate the network’s performance under varying user densities, specifically N∈{5,10,15,20}. Figure 3 encapsulates the algorithm’s capability to handle expanded action spaces. As depicted in Figure 3a, the average reward curves for all user configurations exhibit a steep initial ascent followed by stable convergence. The total system reward naturally scales with the number of users, which fundamentally proves that the decentralized nature of SHIFT-MAB effectively avoids the computational bottlenecks and delayed convergence typically seen in centralized multi-agent systems. Furthermore, the underlying benefit of this scalable architecture is highlighted in Figure 3b. Even as the network becomes highly congested at N=20, the dynamic congestion pricing mechanism successfully prevents resource monopolization, maintaining the final Jain’s fairness index reliably between 0.74 and 0.79. While the total handover count inherently grows with the user population, it remains strictly bounded, demonstrating that the system gracefully accommodates dense deployments without sacrificing equitable resource distribution.

**Figure 4:**
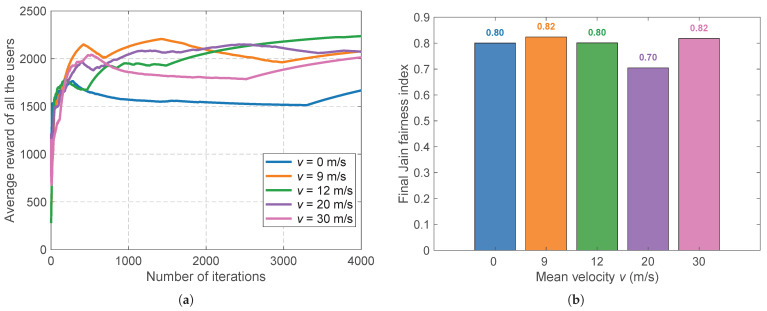
Adaptability and scalability under different mean velocities $\bar{v}$. (**a**) The impact of the user velocity on the average reward convergence, illustrating the benefits of spatial diversity. (**b**) The stability of the final Jain fairness index against varied mobility conditions.

Beyond the network density, the algorithm’s adaptability to highly dynamic temporal environments is examined by varying the mean mobility velocity $\bar{v}\in \left\{0,9,12,20,30\right\}\;m/s$. Figure 4 reveals the system’s robust convergence across diverse mobility profiles. A noteworthy observation from Figure 4a is that intermediate velocities (e.g., 9m/s and 12m/s) achieve slightly higher final rewards than the completely stationary scenario ($\bar{v}=0\;m/s$). This counterintuitive phenomenon occurs because moderate mobility introduces spatial diversity; it acts as an environmental catalyst that physically moves users out of localized deep fading dips, preventing them from being permanently trapped in suboptimal channel conditions. The critical enabler of this benefit is the physical hysteresis gate ($\delta_{\mathrm{hys}}$), which acts as a spatial low-pass filter. It absorbs the rapid, small-scale channel fluctuations induced by mobility, ensuring that spatial diversity translates into throughput gains rather than destructive ping-pong handovers. Consequently, Figure 4b confirms that Jain’s fairness index remains highly resilient (strictly between 0.70 and 0.82) regardless of the velocity, proving that the EWMA sliding window comprehensively tracks non-stationary fading without penalizing highly mobile users.

### 6.3. Robustness Analysis

**Figure 5:**
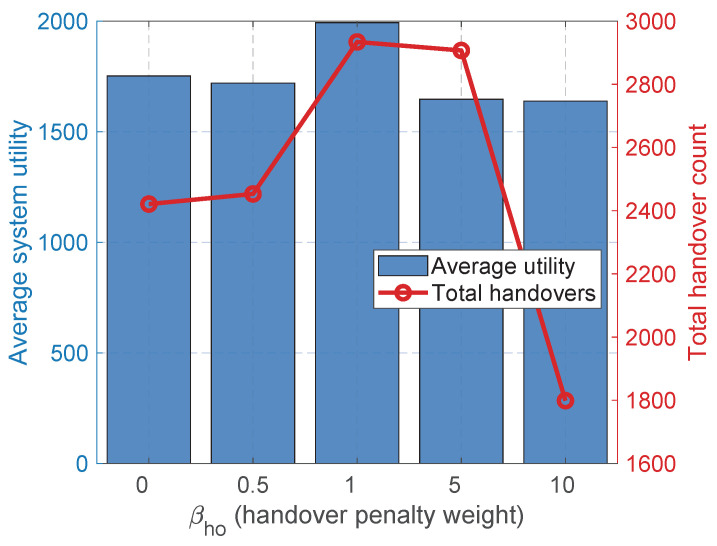
Robustness analysis of the handover penalty weight $\beta_{\mathrm{ho}}$. This explicitly showcases the optimization trade-off: mitigating the total handover count while maximizing the average system utility.

A critical requirement for practical 6G implementation is the algorithm’s robustness to hyperparameter variations. We first investigate the sensitivity of the system to the handover penalty weight $\beta_{\mathrm{ho}}$, sweeping its value across {0,0.5,1,5,10}. Figure 5 illustrates the intrinsic trade-off between the system utility and the frequency of handovers. As the penalty weight increases from 0 to 10, the total handover count drops sharply, confirming the effectiveness of the penalty term in suppressing unnecessary signaling overhead. However, the average utility exhibits a clear U-shaped trend: it peaks at a moderate penalty ($\beta_{\mathrm{ho}}=1$) and degrades significantly when the penalty becomes excessively aggressive ($\beta_{\mathrm{ho}}\geq 5$). In particular, the utility under severe penalties actually falls below the baseline without penalties ($\beta_{\mathrm{ho}}=0$). This counterintuitive phenomenon occurs because an overly strict penalty induces a “handover paralysis” effect. Specifically, UE becomes physically trapped in severely degrading links and is discouraged from switching to closer BSs, despite suffering from catastrophic path loss and a poor SINR. This observation profoundly highlights the necessity of identifying the optimal trade-off point ($\beta_{\mathrm{ho}}=1$), where the penalty is precisely calibrated to suppress invalid ping-pongs without paralyzing essential mobility-driven handovers.

**Figure 6:**
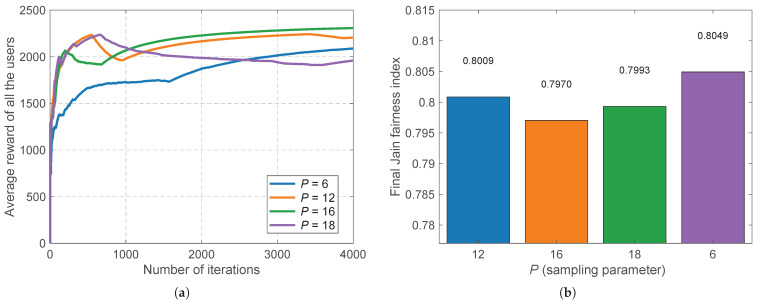
Robustness analysis regarding the sampling parameter *p*. (**a**) Average reward convergence curves highlighting the impact of different exploration budgets. (**b**) The structural resilience of the final Jain fairness index across varied *P* values.

To further validate the stability of the algorithmic learning process, we analyze the sensitivity to the exploration sampling parameter p∈{6,12,16,18}, as depicted in Figure 6. Figure 6a indicates that the learning curves fluctuate slightly during the early exploration phase depending on the budget. As observed in the initial iterations of Figure 6a, the forced pure exploration phase inherently causes a temporary steep dip in the system utility because UE is forced to sample suboptimal BSs. However, this bounded short-term overhead is essential to build accurate channel estimates. Insufficient exploration (p=6) leads to the premature exploitation of suboptimal BSs, while excessive exploration (p=18) wastes time sampling poor links, thereby delaying convergence. Conversely, a mid-range sampling parameter (e.g., p=12 or 16) optimally calibrates the explore–exploit boundary, yielding the highest long-term rewards. Concurrently, Figure 6b demonstrates a profound structural benefit: the final fairness index remains remarkably stable (tightly clustered around 0.80) across all the tested *p* values. This confirms that the fairness-aware reward structure inherently protects minority users and enforces equity, making the system highly robust and largely insensitive to minor inaccuracies in hyperparameter tuning.

Furthermore, while we primarily evaluated $\beta_{\mathrm{ho}}$ and *p*, empirical testing shows that the system performance is also highly sensitive to the hysteresis margin $\delta_{\mathrm{hys}}$ and fairness weight ω. An overly large $\delta_{\mathrm{hys}}$ completely eliminates ping-pong handovers but forces UE to stay connected to suboptimal BSs, slightly reducing the peak throughput. Conversely, a higher ω strictly enforces Jain’s fairness but may degrade the sum rate as more resources are diverted to cell-edge users. Our parameter selection naturally strikes an optimal balance.

To further strengthen the practical deployability of SHIFT-MAB, we provide the following guidance for parameter selection in real-world networks. The hysteresis margin $\delta_{\mathrm{hys}}$ should be dynamically calibrated based on the environment’s shadowing variance and average user velocity. For highly mobile environments with severe fast fading, $\delta_{\mathrm{hys}}$ should be set higher (e.g., 0.5∼0.8) to prevent rapid ping-pong effects, whereas, in static indoor scenarios, a lower value (e.g., 0.1∼0.3) favors prompt associations and throughput maximization. Meanwhile, the fairness weight ω should be tuned according to the service level agreement (SLA) of the network slice. For eMBB slices prioritizing peak sum rates, ω can be relaxed, whereas, for slices requiring strict uniform coverage, a higher ω (e.g., ω≥300) is mandatory to guarantee the QoS of cell-edge users.

### 6.4. Ablation Study and Baseline Comparison

**Figure 7:**
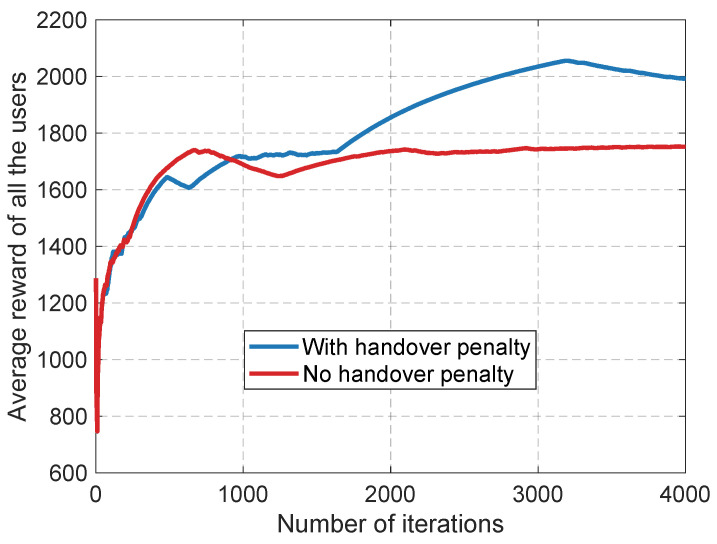
Ablation study demonstrating the critical role of the handover penalty. The penalty ensures stable long-term convergence by preventing the system from collapsing under excessive ping-pong overhead.

Although the sensitivity analysis in Section 5.3 establishes the macroscopic trade-off regarding the penalty weight, we conduct an in-depth ablation study to isolate its impact on the underlying *learning dynamics*. Specifically, we examine the iteration-wise convergence behavior by comparing the optimal framework ($\beta_{\mathrm{ho}}=1$) against a no-penalty baseline ($\beta_{\mathrm{ho}}=0$). As shown in Figure 7, removing the penalty allows the algorithm to experience a slightly faster initial surge in rewards, as agents greedily chase instantaneous SINR peaks. However, this lack of mobility awareness soon triggers a catastrophic chain of excessive ping-pong handovers. The accumulated interruption time and signaling overhead quickly supersede the throughput gains, causing the reward curve to plateau prematurely around 1750. In stark contrast, the inclusion of the handover penalty enforces a more deliberate and far-sighted learning process, ultimately allowing the system to overtake the no-penalty variant and converge stably at a significantly higher long-term utility value (approximately 2000).

**Figure 8:**
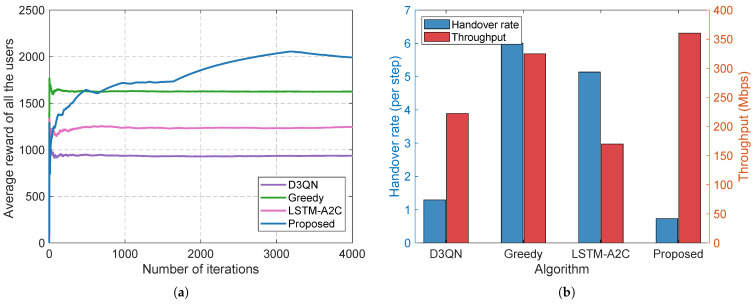
Comprehensive comparison against state-of-the-art RL baselines. (**a**) The learning efficiency and rapid convergence of SHIFT-MAB compared to the sluggish performance of D3QN and LSTM-A2C. (**b**) The optimal handover–throughput trade-off, demonstrating SHIFT-MAB’s ability to maximize data rates while minimizing ping-pong disruptions.

Finally, we comprehensively compare the proposed SHIFT-MAB framework against state-of-the-art reinforcement learning baselines, including D3QN, LSTM-A2C and a greedy heuristic approach. The considerable superiority of the proposed method is explicitly validated in Figure 8. As observed in Figure 8a, standard DRL methods like D3QN and LSTM-A2C severely struggle with the curse of dimensionality and the non-stationary Markov decision processes (MDPs) caused by mobile multi-agent interference. Consequently, they require massive exploration episodes and suffer from suboptimal convergence, stagnating below 1300. Conversely, SHIFT-MAB completely overcomes this bottleneck through its decoupled two-layer bandit structure and lightweight EWMA updates, achieving near-instantaneous convergence and securing an average reward exceeding 2000. More crucially, Figure 8b highlights the ultimate practical metric: the handover–throughput trade-off. The greedy approach blindly maximizes the throughput but incurs a prohibitive handover rate (approaching 6.0 per step), rendering it useless in practice. While DRL baselines manage to lower the handover rate, they suffer from catastrophic throughput degradation. By organically integrating the physical hysteresis gate ($\delta_{\mathrm{hys}}$) to deterministically intercept suboptimal switching, our SHIFT-MAB effectively suppresses the handover rate to an industry-leading minimum (approximately 0.7) while simultaneously unlocking the highest average network throughput among all evaluated schemes.

### 6.5. Discussion and Practical Limitations

While the proposed SHIFT-MAB framework effectively balances fairness and mobility, certain practical limitations must be acknowledged. The framework relies on the periodic broadcast of the global congestion prices $\lambda_{j}^{\left(\cdot \right)}\left(t\right)$ and Jain’s fairness index J(t). Since these are single scalar values, their bandwidth requirement is negligible compared to exchanging high-dimensional model gradients in federated learning. However, ensuring their timely delivery across the network necessitates ultra-reliable low-latency control channels (URLLC). In extremely dense 6G deployments, synchronization latency could temporarily degrade the algorithm’s convergence speed. Future work may explore hierarchical clustering to mitigate this signaling dependency.

## 7. Conclusions

This paper has addressed the joint uplink and downlink user association and resource allocation problem in mobile 6G fully decoupled radio access networks. We have specifically focused on solving three critical intertwined issues: asymmetric interference, persistent network unfairness and mobility-induced ping-pong handovers. To this end, we have designed a new mobility-aware and fairness-guaranteed distributed learning framework, termed Sliding-Window Hysteresis-Integrated Fairness Two-Layer MAB. In this framework, we have constructed a composite handover penalty model with a strict physical hysteresis margin to eliminate redundant signaling overhead, and we have decoupled the original high-dimensional joint optimization problem to circumvent the curse of dimensionality for efficient adaptation to non-stationary wireless environments. Theoretical analyses have demonstrated that the decoupled framework maintains optimality, achieves sublinear regret and ensures fair resource allocation convergence. Simulation results have confirmed SHIFT-MAB’s superior performance in terms of the adaptive tuning of the handover penalty and rational use of the hysteresis mechanism, delivering an optimal throughput–handover trade-off, along with strong robustness in high-velocity mobility scenarios and scalable fairness in dense user deployments. Future research will extend this framework by integrating multi-agent learning with partial observability and reconfigurable intelligent surfaces (RIS), aiming to further enhance mobile coverage and network performance in complex 6G scenarios.

## Figures and Tables

**Table 1:** Comparison of existing approaches and our proposed SHIFT-MAB.

Reference	Architecture	Algorithm	Handover Handling	Mobility Adapt	Fairness
Qian et al. [27]	FD-RAN	Matching and SCA	N/A	Low	No
Lee et al. [25]	Cog. Radio	Absorbing MC	Cost-based	Moderate	No
Manalastas et al. [10]	5G	XGBoost	Supervised	N/A	No
Zhao et al. [28]	HetNet	D3QN	Implicit (Penalty)	Low	QoS-aware
Wang et al. [16]	Cellular	A3C	Implicit (Penalty)	Moderate	No
Zhang et al. [29]	FD-RAN	Multi-Agent DRL	Implicit (Penalty)	Moderate	No
Chen et al. [32]	FD-RAN	Stepwise RL	N/A	Moderate	Subjective
**Our Work**	**FD-RAN**	**SHIFT-MAB**	**Explicit ($\delta_{\mathrm{hys}}$)**	**High**	**Jain’s index**

**Table 2:** List of key notations.

Notation	Definition
N	Set of mobile UE
$M^{\left(\cdot \right)}$	Set of BSs in UL/DL direction
$K^{\left(\cdot \right)}$	Set of channels in UL/DL direction
$P^{\left(\cdot \right)}$	Discrete power level set in UL/DL direction
$b_{i,j,k}^{\left(\cdot \right)}\left(t\right)$	Binary association indicator for UE *i* on BS–channel pair (j,k)
$p_{i,j,k}^{\left(\cdot \right)}\left(t\right)$	Transmit power level of UE *i* on BS–channel pair (j,k)
$s_{i,t}^{\left(\cdot \right)}$	Serving BS index for UE *i* at time *t*
$\chi_{i,t}^{\left(\cdot \right)}$	Handover indicator for UE *i* at time *t*
$h_{i,j,k}^{\left(\cdot \right)}\left(t\right)$	Channel gain from BS *j* to UE *i* on channel *k*
$\Gamma_{i,j,k}^{\left(\cdot \right)}\left(t\right)$	SINR for UE *i* at BS *j* on channel *k*
$W^{\left(\cdot \right)}$	Channel bandwidth in UL/DL direction
$N_{0}^{\left(\cdot \right)}$	Noise power spectral density in UL/DL direction
$r_{i,j,k}^{\left(\cdot \right)}\left(t\right)$	Achievable rate of UE *i* on BS–channel pair (j,k)
$E_{h,i}^{\left(\cdot \right)}\left(t\right)$	Composite handover cost for UE *i* in direction (·) at time *t*
$\lambda_{j}^{\left(\cdot \right)}\left(t\right)$	Dynamic congestion price at BS *j*
J(t)	Jain’s fairness index at time *t*
$P_{\max}^{\left(\cdot \right)}$	Maximum power budget limit in UL/DL direction
$C_{j}^{\left(\cdot \right)}$	Capacity limit of simultaneous associations for BS *j*
$\delta_{\mathrm{hys}}$	Hysteresis margin for handover trigger
$\beta_{r}$	EWMA smoothing factor for rate and mean estimates
$J_{\min}$	Minimum required time-averaged Jain’s index
$\omega ,\beta_{\mathrm{ho}}$	Fairness reward weight and handover penalty weight
ρ	Temporal discount factor
α	Memory parameter in the Gauss–Markov mobility model
Ω	Path loss at the reference distance
κ	Path loss exponent

**Table 3:** Experimental parameters.

Parameter	Value/Range
*System parameters*	
Number of users (*N*)	5 (default)
Base stations ($M^{U}$, $M^{D}$)	3
Channels ($K^{U}$, $K^{D}$)	3
Power levels (*L*)	5 (0.5–2.5 W per level)
Coverage area (horizontal)	100m×100m
BS height/UE height	25 m/1.5 m
Mobility	Gauss–Markov; $\bar{v}$, Δt=1 s
Path loss exponent	2
Noise PSD	−174 dBm/Hz
Bandwidth (*W*)	20 MHz
*Algorithm parameters*	
Handover penalty ($\beta_{\mathrm{ho}}$)	1 (default)
RRC signaling energy ($E_{\mathrm{sig}}^{\left(\cdot \right)}$)	0.1 Joules (UL/DL)
Handover interruption time ($T_{\mathrm{ho}}^{\left(\cdot \right)}$)	0.05 (UL/DL)
Sampling parameter (*P*)	6 (default)
Fairness weight (ω)	300
UL-DL weight (γ)	0.5
Candidate budget	200; neighbor sampling
Price step $\eta_{0}$/cap $\lambda_{\max}$	10/5
EWMA factor ($\beta_{r}$)/hysteresis ($\delta_{\mathrm{hys}}$)	0.8/0.5
*Varied scenarios in experiments*	
Exp. 1 (scalability)	N∈{5,10,15,20}
Exp. 2 (velocity)	$\bar{v}\in \left\{0,9,12,20,30\right\}$ m/s
Exp. 3 (trade-off)	$\beta_{\mathrm{ho}}\in \left\{0,0.5,1,5,10\right\}$
Exp. 4 (*P*-sensitivity)	P∈{6,12,16,18}
Exp. 5 (ablation)	Handover penalty ($\beta_{\mathrm{ho}}=1$ vs 0)
Exp. 6 (RL comparison)	Proposed, D3QN, Greedy, LSTM-A2C

## Data Availability

The data presented in this study are available on request from the corresponding author.
